# Designing super-fast trimodal sponges using recycled polypropylene for organics cleanup

**DOI:** 10.1038/s41598-023-41506-6

**Published:** 2023-08-29

**Authors:** Junaid Saleem, Zubair Khalid Baig Moghal, Gordon McKay

**Affiliations:** 1 Division of Sustainable Development, College of Science and Engineering, Hamad Bin Khalifa University, Qatar Foundation, Doha, Qatar; 2 Center for Advanced Materials, Qatar University, Doha, Qatar

**Keywords:** Environmental chemistry, Environmental impact, Engineering, Materials science

## Abstract

Sorbent pads and films have been commonly used for environmental remediation purposes, but designing their internal structure to optimize access to the entire volume while ensuring cost-effectiveness, ease of fabrication, sufficient strength, and reusability remains challenging. Herein, we report a trimodal sorbent film from recycled polypropylene (PP) with micropores, macro-voids, and sponge-like 3D cavities, developed through selective dissolution, thermally induced phase separation, and annealing. The sorbent has hundreds of cavities per cm^2^ that are capable of swelling up to twenty-five times its thickness, allowing for super-fast saturation kinetics (within 30 s) and maximum oil sorption (97 g/g). The sorption mechanism follows a pseudo-second-order kinetic model. Moreover, the sorbent is easily compressible, and its structure is retained during oil sorption, desorption, and resorption, resulting in 96.5% reuse efficiency. The oil recovery process involves manually squeezing the film, making the cleanup process efficient with no chemical treatment required. The sorbent film possesses high porosity for effective sorption with sufficient tensile strength for practical applications. Our integrated technique results in a strengthened porous polymeric structure that can be tailored according to end-use applications. This study provides a sustainable solution for waste management that offers versatility in its functionality.

## Introduction

Plastics have become indispensable due to their wide range of uses and significant physical characteristics, which play a crucial role in the modern economy^1,2^. However, the production and disposal of plastic waste have had a severe impact on the environment. It is estimated that billions of tons of plastics have been produced to date, and the progressive accumulation of plastic waste over the past few decades has attracted significant attention^3,4^.

Traditional methods of plastic waste treatment have their limitations. The Landfill^5^ and incineration^6,7^ are major sources of toxic gases, as well as micro-pollutants^2,8^. To address this problem, various solutions have been proposed for recycling plastic waste^9^ to ensure sustainable use of fossil resources and protect the environment. These solutions involve mechanical recycling, thermo-chemical recycling, and upcycling. Mechanical recycling is widely used for large plastic waste but is limited due to additive contamination, material degradation, limited range of materials, and processing complexity^10–12^. Furthermore, pyrolysis is recently considered as a promising method for thermo-chemical recycling of plastic waste. However, it may not always be a feasible solution due to its high energy consumption, production of toxic byproducts, and variable quality of the resulting products^3,13,14^.

Upcycling of plastic waste refers to the process of transforming discarded plastic materials into new products with higher value and functionality. Some unconventional approaches to polymer upcycling include solvolysis, mechanochemistry, photo-reforming, biotechnology, pyrolysis, and dissolution/precipitation methods^15^. Upcycling plastic waste is a better solution compared to other recycling due to higher quality of recycled products, environmental and economic benefits, and greater energy efficiency^15,16^. It yields a diverse range of high-value products, such as photovoltaics, battery electrodes, carbon nanotubes, membranes, additives for reinforcement, as well as fibers, films, and composites, showcasing the potential for transforming waste into valuable resources and promoting sustainable practices^17–23^. Upcycling plastic waste into oil sorbent can also be a useful application, as it can help to mitigate the negative impacts of oil spills on the environment by effectively absorbing the spilled oil^1,24^.

While sorbents are commonly used for oil spill applications, there is still room for improvement, especially in how they are designed^25,26^. To be effective in oil–water emulsion, an oil sorbent needs to have specific characteristics: it must be hydrophobic, porous, and oleophilic. Porous oil sorbents allow oil to pass through when they come into contact with oil and water mixtures. A hydrophobic surface allows the sorbent to absorb more oil because it repels water and attracts oil^27,28^. The sorption mechanism depends on various factors, including the porous structure, the sorbent's attraction towards oil, the surface's roughness, the cohesion between oil molecules on the surface and inside the pores due to capillary action, and the adhesion between the sorbent's surface and oil molecules^26,29^.

Several sorbents comprise natural inorganic products such as silica, zeolites, clay, calcium, etc.^26^ or biodegradable organic materials like corncob, sugar cane bagasse, straw, wood fibers, and cotton fibers^30,31^. However, natural oil sorbents tend to have limited oil uptake capacity with relatively higher water uptake^26,32^. To improve the oil uptake capacity, synthetic sorbents have been used. They include melt-blown fiber-based booms and pads made of polypropylene or relatively thick sheets fabric-stitched and melt-spun polymers^32^. Yet, these sheets are not applicable for thin water-borne oil films as a portion of these sheets goes into the water, thereby hindering the oil sorption capacity.

Graphene aerogels provide high uptake values but are extremely light and fragile, making them almost impossible to handle by end users. Moreover, they are not recyclable by simple mechanical squeezing. Instead, they require chemical treatment to remove oil trapped inside the 3D porous structure^33^. Further, the process of converting graphite to graphene increases the overall cost of production. Carbon nanotubes^34^ have also been used to utilize high surface area, but the cost of production is extremely high to be used for commercial applications. Aerogels based on polymers such as polypropylene (PP), polyvinylidene fluoride (PVDF), polycarbonate (PC), high-density polyethylene (HDPE), polyurethane (PU) were also reported with limited oil uptake capacity ranges from 6 to 25 g/g^33,35–38^. Aerogels made up of plastic waste have also been produced. In one such example, waste polyethylene terephthalate (PET) based aerogel was prepared, but it was reported that it had limited recyclability due to the removal of silane coating during the squeezing^39^.

Hence, choosing the right type of material coupled with an optimized internal structure is imperative to make super-fast, super-oleophilic, reusable, and cost-effective sorbents. In this respect, polypropylene is amongst the best precursors for preparing super oil-sorbent. It is more porous, hydrophobic, and oleophilic than other polyolefins. Compared with graphene and carbon nanotubes, polypropylene-based pads, sheets, films, and aerogels are more flexible, reusable, and cheaper. Commercial sorbent pads are mostly made of polypropylene^26^, such as 3M-HP-255, 3M-156, and Chemtex-BP-9W. These pads are produced by spinning polymer using heat and air into long fluffy fibers or threads, followed by pressing the fibers together between hot rollers, thereby obtaining a flexible solid fabric. Yet, they are not applicable for thin water-borne oil films as a portion of these pads go into the water, thereby hindering the oil sorption capacity. Further, these commercial pads are for one-time use only as oil gets trapped in the internal structure, and reusing them will significantly decrease the oil uptake capacity, making them unsustainable. Lastly, though they are used to clean up oil spills, they indirectly cause further plastic pollution due to their one-time application. Hence, there is a need to prepare an oil sorbent that can absorb a higher amount of oil without absorbing water, sustainable and reusable.

Multiple investigations have documented the successful utilization of plastic waste as sorbents and membranes for oil–water separation^40–45^. Separators for oil–water emulsions have been fabricated using: PET waste via electrostatic spinning, in-situ deposition and surface modification^46^, Biomimetic fabrication of PET waste via electrospinning with enhanced stability and demulsibility^47^, kelvar fiber waste via combining solvent replacement and freeze-drying route^48^, and PE waste via swelling, solvent extraction and freeze-drying^20^. As these examples demonstrate, plastic waste is a viable feedstock for oil sorbent production.

The present work focuses on synthesizing a sustainable trimodal oil sorbent consisting of micropores, macro-voids, and sponge-like 3D cavities using waste polypropylene to overcome the problems discussed above. The purpose of creating this trimodal structure is to improve and enhance the oil sorption properties. Macro-voids and micropores accelerated oil sorption saturation kinetics, while 3D cavities improved oil retention. The interaction of micropores, macro-voids, and swellable cavities results in high oil absorption values, which can reach up to 97 times the mass of the sorbent. Furthermore, the prepared sorbent is considered a sustainable material since it maintains its structure even after oil sorption, desorption, and resorption, and can be recycled with 97% efficiency for tens of cycles. Additionally, the oil can be quickly extracted from the film through mechanical squeezing, without the need for chemical processing, ensuring an eco-friendly process. The sorbent film has both the required porosity for efficient sorption and adequate tensile strength for real-world applications.

## Experimental section

### Materials

Plastic waste was collected from the landfill site and was then washed in detergent water to remove debris and unwanted particles. The cleaned PP waste was then shredded into fine pieces for solution preparation. As a solvent, an isomeric xylene mixture was used without further purification. Sodium chloride (NaCl), common table salt, was purchased from a local store and sieved to 150–200 μm particle size for cavity formation.

#### Swellable cavities formation

The dissolution technique coupled with spin-coating were used to prepare trimodal sorbent. Dissolution/precipitation is a promising technique for recovering polymers from plastic waste, removing additives and producing pure (cystallized) polymers^49^. Waste PP was dissolved in a 10 ml isomeric xylene solution per each gram at 130 °C in a closed environment to avoid solvent loss, followed by the addition of 2 g of NaCl as a cavity forming agent or filler. The solution was stirred for at least 20 min using magnetic stirrer until the solution is homogenized and the insoluble filler was completely dispersed. The hot solution was then poured onto the heated glass substrate, at a temperature below the boiling point of the solvent to prevent the early solidification of the polymer, and spin coated with gradual speed from 400 rpm for 10 s up to maximum of 3000 rpm for 120 s to achieve a uniform thickness.

#### Annealing and washing

**Figure 1:**
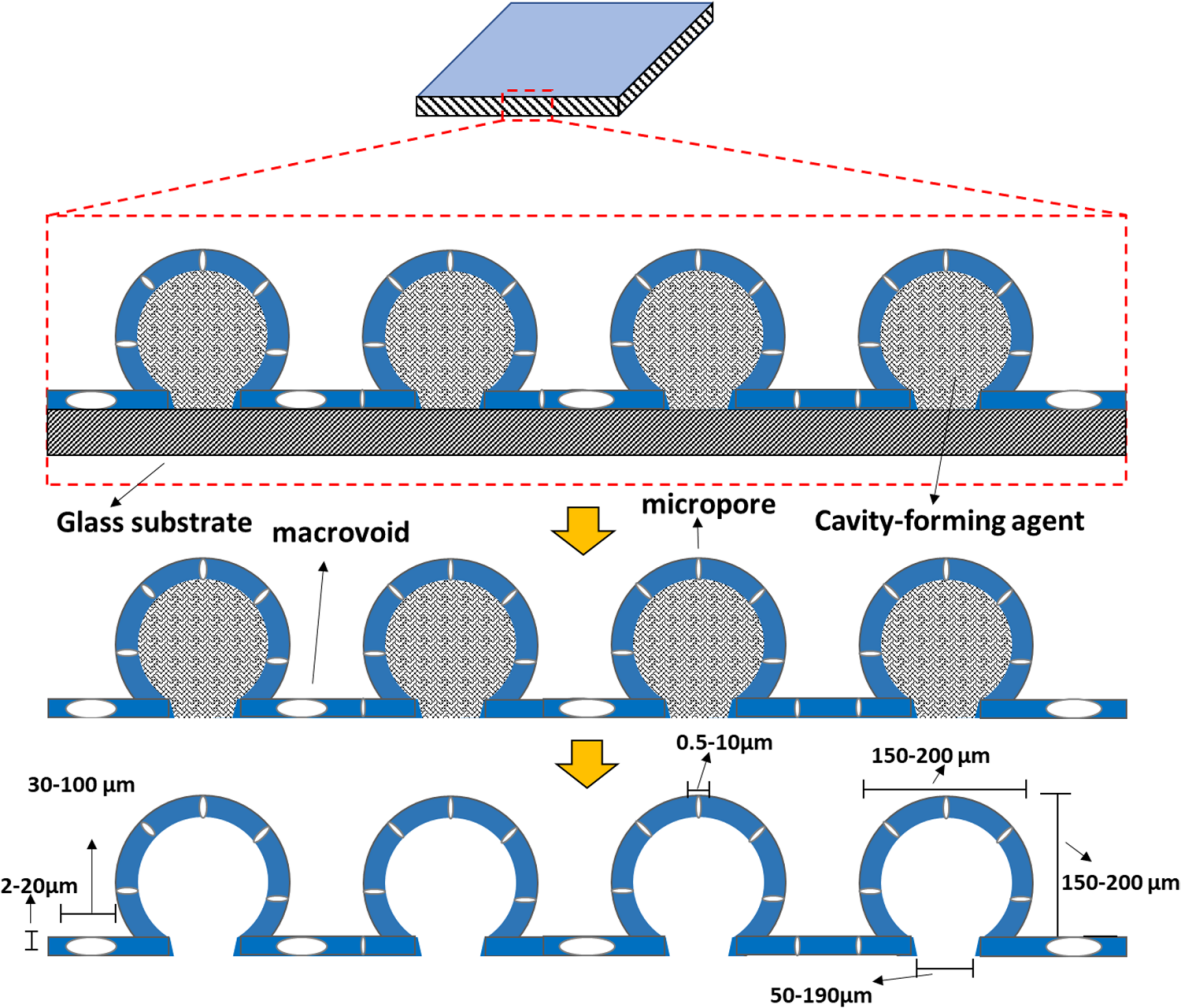
Visual representation of creating micropores, macro-voids, and cavities.

**Figure 2:**
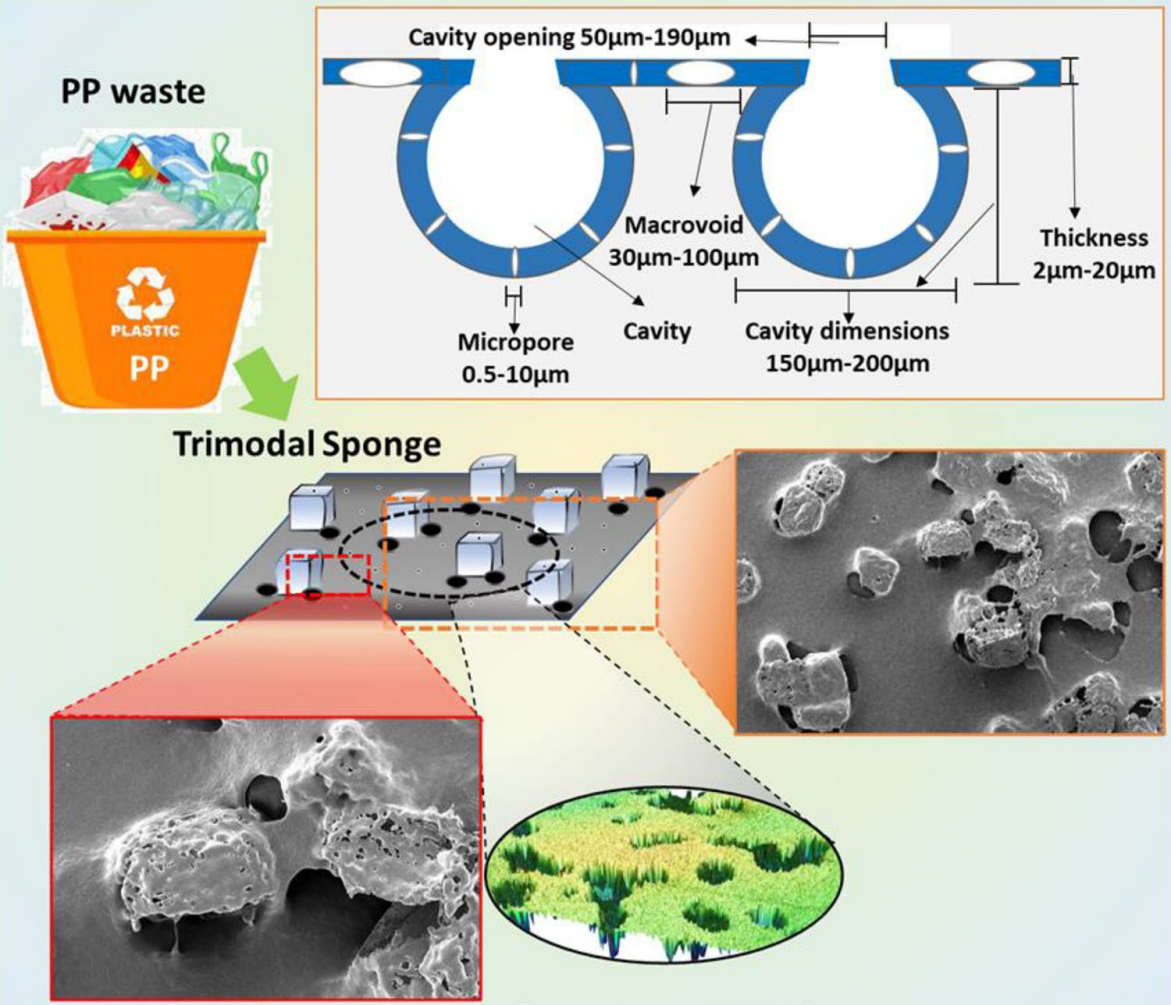
Recycled PP-based trimodal sponge sorbent.

The glass substrate with the as-prepared thin film was then heated at a temperature near melting of the polymer for an optimum time in a hot air oven to peel off the sample but without gel formation and kept at room temperature for cooling. The cooled sample was then washed with water to filter out the cavity forming agent from the thin film. Figure 1 presents a visual representation of trimodal sponge preparation, while Fig. 2 shows the structure of the sponge.

#### Sorption capacity

A variety of sorbents with different film thicknesses and annealing conditions were examined for oil sorption ability, each with a surface area of 25 cm^2^. The sorbent was immersed in the sorbate for 5, 10, 20, 30, and 60 s and was removed with a tweezer and weighed for immediate measurements. It is then left to hang for a predetermined amount of time, which allowed it to reach equilibrium conditions as the sorbate drains. At this equilibrium condition, the final uptake capacity was quantified. Both the measurements were used to model the sorption kinetics behaviour. Additionally for dripping kinetic, the sorbent was poured into the sorbate until saturation was obtained, and then it was permitted to hang for 30, 60, 120, 300, and 900 s, respectively. The amount of sorbate determines the ability to absorb oil that a unit mass of dry sorbent can absorb. Moreover, the sorbent's reusability was tested for resorption by means of solvent washing and mechanical squeezing to investigate the recyclability.

## Results and discussion

### Creation of trimodal structure

#### Factors affecting cavity size and opening

*S*ize of the cavity opening can be influenced by several factors, including: (a) Preheating of the solid substrate is required for large cavity openings, whereas small cavity openings do not require preheating, (b) The contact area between the cavity forming agent and the solid substrate should be high for large cavity openings. This can be achieved using a cubic or cuboid-shaped cavity forming agent. For small cavity openings, the contact area should be small, which can be achieved by using a spherical-shaped cavity forming agent, and (c) The viscosity of the polymer solution should be high for large cavity openings and low for small cavity openings.

#### Factors affecting the size of macro-voids

The size of the macro-voids can be affected by the following factors: (a) A small ratio of polymer to cavity forming agent is necessary for larger macro-voids, while a high ratio is required for smaller macro-voids, (b) The speed of rotation should be high for larger macro-voids and low for smaller macro-voids, and (c) The viscosity of the polymer solution should be low for larger macro-voids and high for smaller macro-voids.

#### Role of rpm on the formation of macro-voids and cavity openings

The formation of macro-voids and cavity openings can be influenced by the rpm (revolutions per minute). The recommended rpm ranges for different scenarios are: (a) For macro-voids with large cavity openings, the rpm should range from 600 to 900 with a preheated solid substrate, (b) For macro-voids with small cavity openings, the rpm should range from 600 to 900 without a preheated solid substrate, (c) For large cavity openings with no macro-voids, the rpm should range from 300 to 500 with a preheated solid substrate, and (d) For small cavity openings with no macro-voids, the rpm should range from 300 to 500 without a preheated solid substrate.

#### Annealing

Annealing was performed to prevent the collapse of the film structure due to its weak strength. During annealing, the polymer chained soften, realign, and restructure themselves. This leads to a more compact and dense structure with enhanced crystallinity, which ultimately increased the intermolecular dispersion forces. As a result, the film maintained its structural integrity. Optimum temperature and time of annealing were required to prevent polymer fluidization which might result in pores and voids collapsing. The melting induces the polymer chains to disorder and then reorganize themselves upon cooling under atmospheric conditions.

### DSC, XRD, and FTIR

**Figure 3:**
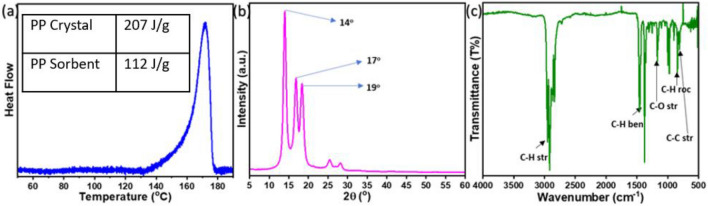
(**a**) DSC spectra of PP, (**b**) XRD spectra of PP, (**c**) FTIR spectra of PP.

DSC was used to used to calculate % crystallinity through enthalphy change calculations as shown in Fig. 3a. The melting peak for PP was observed at around 170 °C and expectedly only one distinguished peak was found, confirming the presence of only PP. The enthalpy was found to be 112.32 J/g, which was compared with 100% pure crystalline PP^50^. The as-prepared sorbent showed 54.2% crystallinity, which shows the semi-crystalline nature of the polypropylene. Figure 3b shows the XRD results of the PP sample. The characteristic peaks for PP were found to be at 14°, 17° and 19°, respectively. Moreover, the crystallinity from the XRD was calculated and was found to be 58%. The XRD outcome of semi-crystalinity support the DSC results.

FT-IR was used to examine the functional groups, as shown in Fig. 3c. At 2836, 2869, 2919, and 2951 cm^-1^, C–H symmetric and asymmetric stretching peaks were observed. While at 1459 and 1376 cm^-1^, C–H symmetrical bending peaks were evident for PP. We observed a C–O peak at 1240 cm^-1^. This peak is due to ether linkage that creates crosslinking between polymer chains and provides strength. The fingerprint region of the FTIR spectra showed the stretching and rocking peaks of C–C and C-H bonds.

### SEM, tensile strength, and contact angle

**Figure 4:**
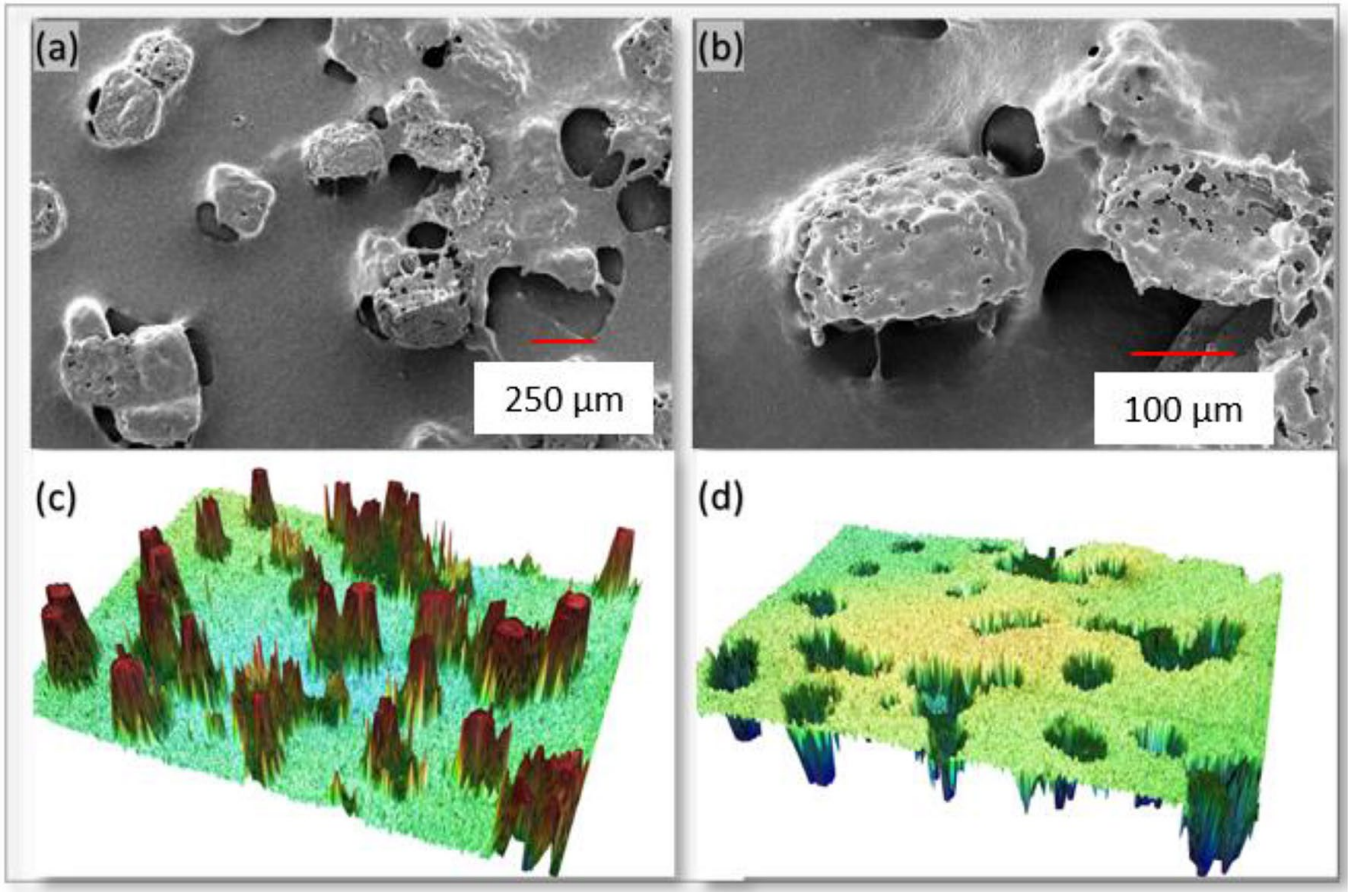
SEM images of PP-based thin film sorbent (**a**) 3D cavities and macrovoids (**b**) close view showing micropores and macro-voids; 3D profilometry imaging of (**c**) top view and (**d**) bottom view of sorbent material.

The SEM images exhibiting cavities, macro-voids, and micropores are shown in Fig. 4a,b. In these images, the PP can be viewed as a network resembling a fibrous web. The sizes of 3D swellable cavities and macro-voids are approximately 50–70 μm and 150–200 μm, respectively (see Fig. 4a). These swellable cavities enhance the sorption volume, and the size of cavities is controlled by the cavity forming agent (NaCl) size. The agent was properly mixed in the solution for even distribution of the cavities within the thin film, which is also evident by the SEM images. In addition, it was found that the glass substrate's temperature significantly influences how big the cavities' openings are. The macro-voids present in the thin film contribute to the sorption capacity as well as the oil diffusion and faster saturation. The dense micropores (~ 5 μm) can be seen in the close view (Fig. 4b), which facilitates oil sorption and oil retention due to cohesive and adhesive. These micropores also improve the retention capacity because of capillary action forces. Thus, the developed film is highly porous to accommodate high retention volume.

Moreover, the 3D-image projection of the cavities and macro-voids was obtained using profilometry (Fig. 4c,d). The 3D shapes of cavities are evident from the top and bottom views. The surface roughness facilitates oil sorption and retention. The oil from the cavities can be collected when the sorbent is mechanically compressed or washed with the solvent, and the sorbent is then utilized for another cycle. The structure of cavities contracts with compression to the thickness of the thin film, which retains its original inflated structure upon reuse and resorption. As a result, these cavities serve as expandable reservoirs.

We investigated the tensile strength of sorbent films at various thicknesses, heating temperatures, and porosity. We employed an inorganic filler as a cavity forming agent with a particle size of 150–200 μm, with a 2:1 concentration of filler to polymer. The target was to develop a sorbent film that possesses both high porosity for effective sorption and sufficient tensile strength for commercial use.

The results showed that the highest porosity of 82% was achieved at a temperature of 25 °C and a film thickness of 16 μm. However, the PP film was not in the form of a freestanding thin film, so the tensile strength was not determined. As the temperature increased to 150–160 °C, the porosity and film thickness decreased due to the condensing and close arrangement of the polymer chains. At 165 °C, a film thickness of 5 μm was obtained with good tensile strength of 10 MPa, but the porosity was less than 1%, which is not desirable for sorption.

Therefore, the optimal conditions were found to be at 160 °C and 20 min, resulting in a 7 μm sorbent film with 34% porosity and a tensile strength of 5 MPa. This film had a good sorption capacity and was strong enough to be used as a freestanding thin film. Table 1 presents the effect of annealing temperature and annealing time on porosity and strength.

**Table 1:** Effect of annealing temperature and annealing time on porosity and strength.

SN	Porosity (%)	Annealing temperature (^o^C)	Annealing Time (min)	Strength (MPa)
1	82	25	0	ND
2	60	150	25	1
3	34	160	20	5
4	9	160	25	8
5	< 2	165	5	10

**Figure 5:**
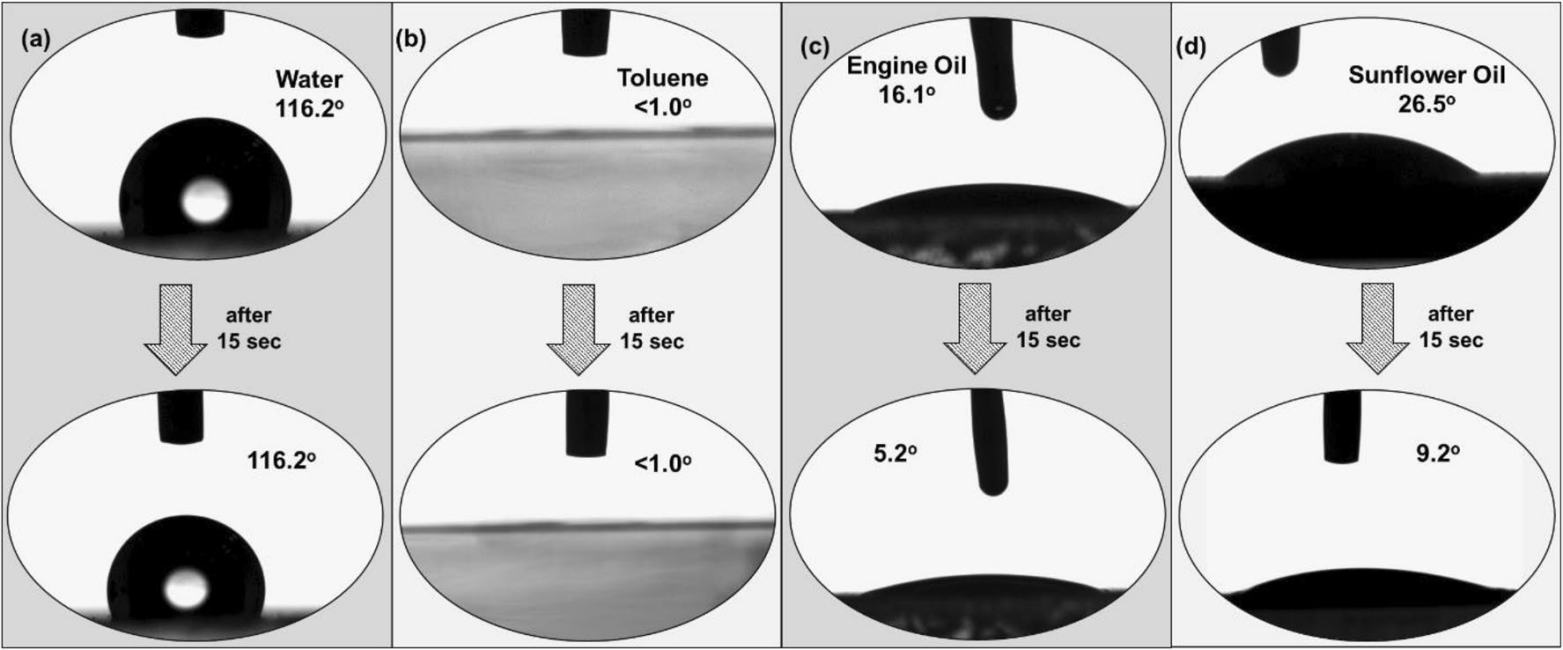
Sorbent film’s contact angle measurements for (**a**) water, (**b**) toluene, (**c**) engine oil, and (**d**) sunflower oil, at 0 s and 15 secs.

The main application of the developed PP sorbent is to remove a thin layer of oil from the water surface. For this purpose, the sorbent material should possess hydrophobic properties. Therefore, contact angles were measured for water, engine oil, toluene, and sunflower oil, as shown in Fig. 5a–d. It was found that the contact angle is 116.2° for water, which was desired. For toluene, engine oil, and sunflower oil, the contact angles were found to be < 1°, 16.1°, and 26.5°, respectively. After 15 s, the contact angles were measured, where we observed a rapid decrease in the contact angle, which was attributed to the fast spreading of fluid over the sorbent surface, sorption inside the surface, and helped in quick penetration of fluid into the porous structure.

### Organic sorption

The cavities, macro-voids, and micropores in the thin film sorbents allowed for substantial oil retention volume and oil penetration. The cohesion and adhesion forces between oil molecules in the sorbent's structure and on its surface, as well as the adhesive forces between the sorbent and oil, were responsible for the improved oil retention capacity. These sorbents are inert in nature, do not degrade when used for oils, and can be recycled and reused.

The oil was immediately adsorbed onto the surface as it came in contact with the thin film sorbent. When the oil permeated the sorbent and the cavities over time, the absorption capacity increased.

**Figure 6:**
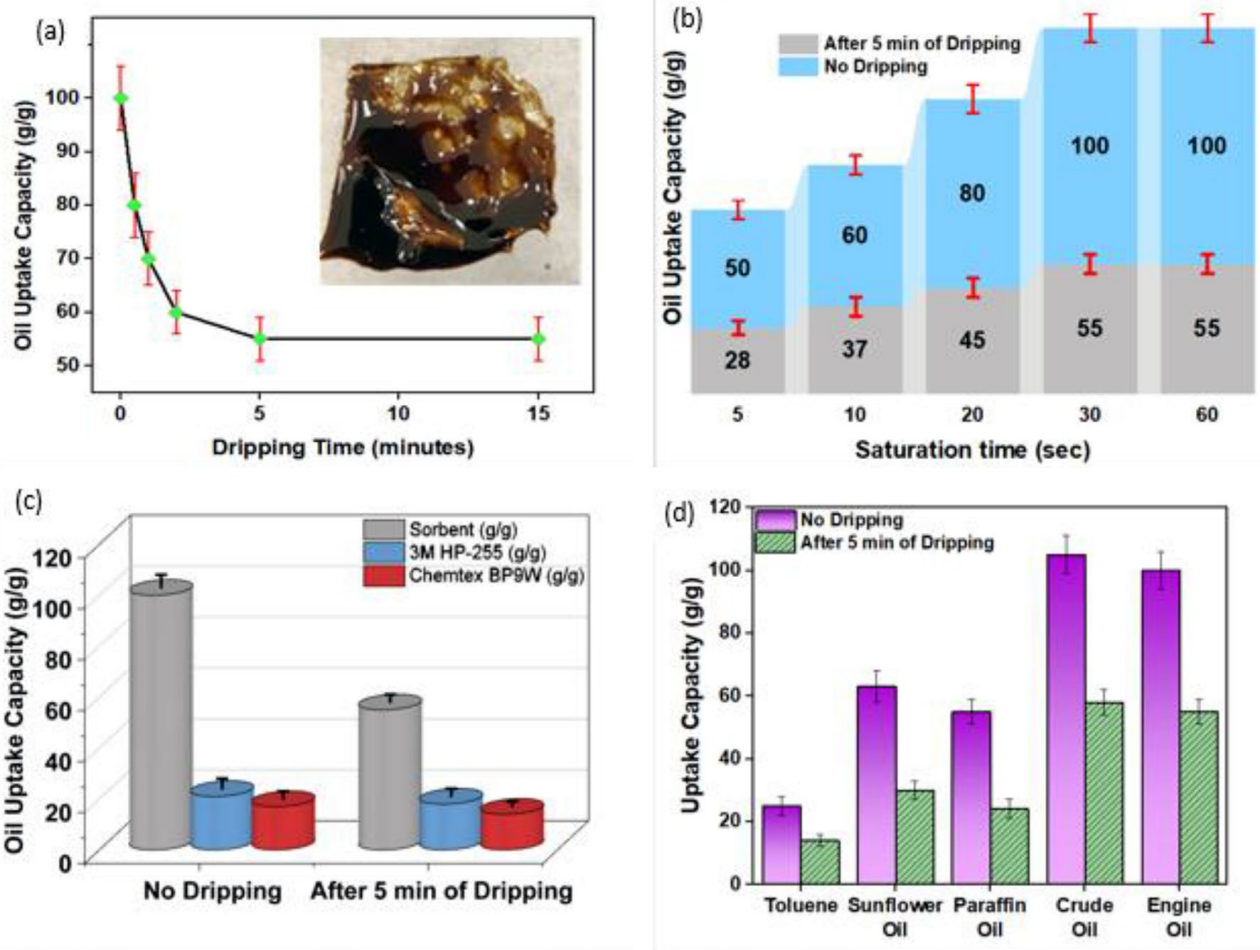
(**a**) Dripping kinetics exhibiting sorption capacity using engine oil (inset, sorbent after oil sorption), (**b**) sorption capacity with time for immediate and equilibrium conditions using engine oil, (**c**) performance comparison of PP sorbent with commercial sorbents, and (**d**) uptake capacity for different oils. The experiment was repeated five times and the standard deviation was reported as the error bars.

The dripping kinetics of PP sorbent is shown in Fig. 6a. The as-prepared sorbent followed an uptake mechanism which was a combination of the following: (a) cohesion between oil molecules trapped inside the internal structure of the oleophilic film (pores, macro-voids, and 3D-cavities) and on the surface, resulting in a strong capillary action; (b) adhesion between oil and sorbent molecules, such that higher viscous oil tended to adhere more at the rough surface; and (c) higher surface area to thickness ratio, providing maximum available sites for oil uptake. A combination of adhesion, cohesion, and a large internal surface area plays a significant role in the oil uptake and retention mechanism^51^. The immediate capacity of sorbent reached a maximum of 97 g/g, and as the dripping started, the capacity decreased and reached the equilibrium state of 52 g/g.

To find out how quickly the oil sorbent reached its maximal absorption capability, the saturation kinetics of the sorbent was studied, as shown in Fig. 6b. The trimodal structure of the sorbent allows absorbing the oil quickly as it reaches maximum capacity in 30 s for both immediate and equilibrium conditions. The saturation kinetic capabilities showed no noticeable change, much as the dripping kinetics. Furthermore, the sorbent was compared with the commercial sorbents, available as pads in Fig. 6c. It can be seen that the sorption capacity of the PP sorbent is around three times that of the commercial sorbents. Moreover, our sorbents are better suited for removing thin oil layers from water.

Figure 6d shows the performance comparison of our sorbent for toluene, sunflower oil, paraffin oil, crude oil, and engine oil. With different oils, differing uptake volumes can be observed; this is because the oils have different viscosities and affinities with the sorbents. The sorbent was found to have a higher uptake capacity for engine oil and crude oil. The oleophilic nature of the sorbents and the 3D-structural design of the cavities are responsible for the good uptake capacity of organic solvents like toluene demonstrated despite their lower viscosity. A total of 55 independent films were used for these experiments. The experiment was repeated five times and the standard deviation was reported as the error bars.

#### Oil–water separation efficiency

**Figure 7:**
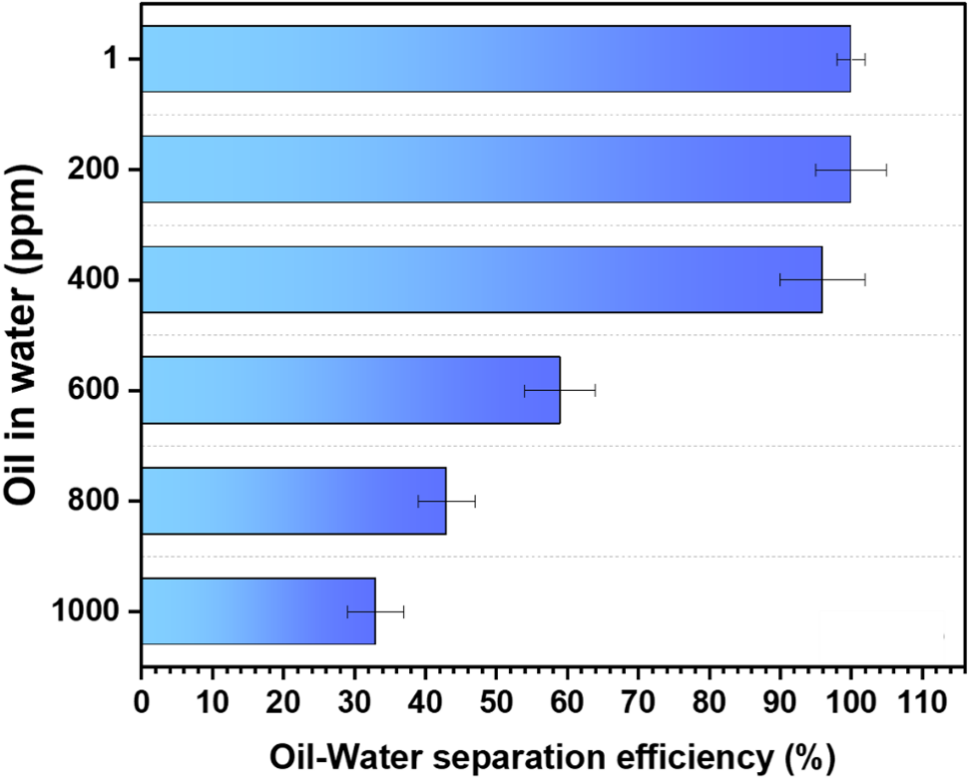
Sorbent efficiency for different oil concentrations in water.

Oil spills on the water surface make it difficult to collect only oil by repelling water; we looked into oil–water separation experiments to understand the effectiveness of the sorbents in practical applications. The selective separation demonstrated strong oil selectivity over water and can be utilized to adsorb and separate oil from water, as shown in Fig. 7. These films have a stronger affinity for oil than for water because they are superoleophilic and hydrophobic. They only absorbed oil and repelled water when placed on thin oil coatings that were placed on the water. A 100 ml volume of water containing various oil concentrations was taken, and a 25 cm^2^ thin film sorbent is placed on the surface. Up to 400 ppm of oil concentration, the separation efficiency was more than 95%. The capacity of the sorbent to absorb water decreased as the percentage of oil in the water increased. This issue can be solved by using a larger sorbent. Nonetheless, the oil selectivity was over 99.5%, and the water retention was less than 0.5%.

### Recyclability

**Figure 8:**
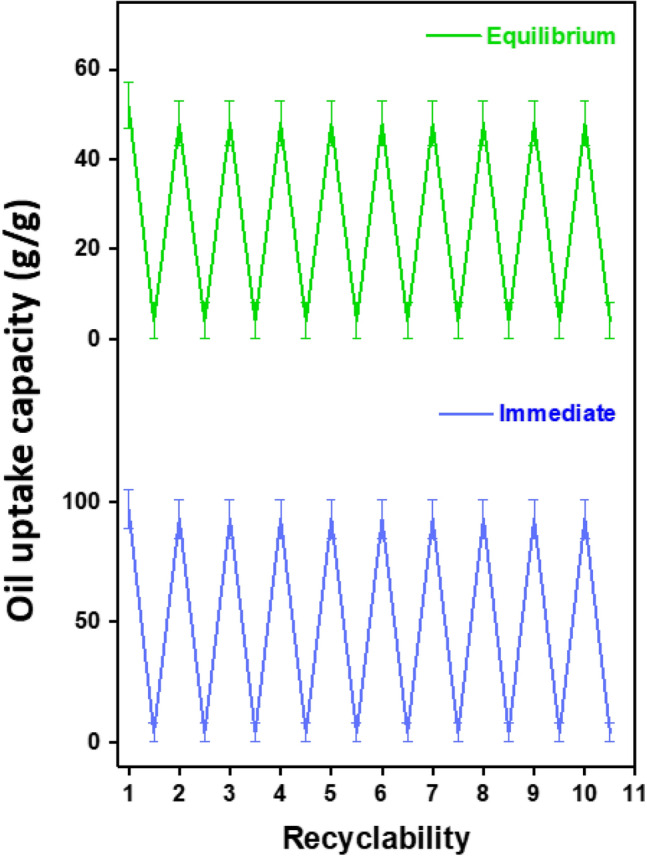
Recyclability of PP-based sorbent for immediate and equilibrium conditions.

To determine whether the sorbent may be reused, the recyclability test was conducted. The sorbents must be strong enough to be reused repeatedly without losing their ability to absorb the material and without causing the structure to collapse. The sorbent thin films were annealed following spin coating. After the sorption and desorption of oil from the sorbent structure, the annealing procedure aided in increasing the mechanical strength of the thin film, making the sorbent a reusable product. The recyclability test was carried out for ten cycles for both immediate and equilibrium conditions, as shown in Fig. 8.

Both mechanical squeezing and solvent washing have been utilized to reuse the sorbent films. The mechanical squeezing technique is the most effective for recycling the sorbent, but a small amount of oil is trapped inside the material since pressing won’t be enough to get rid of oil from the pores. When the sorbent is reused, the thin film cavities retain their structural integrity and absorb the oil to their maximum capacity. Hence, mechanical squeezing facilitates oil collection easily and rapidly, with a 97% efficiency. On the contrary, the solvent-washing approach takes a lot of time but is completely effective in terms of oil recycling. This procedure involves squeezing the oil after oil sorption and submerging the sorbent in hexane which dissolves the oil and thoroughly cleans the sorbent. Furthermore, the sorbent film's maximum capacity for absorption was effectively utilized after being reused. Finally, the oil and hexane were separated by evaporating the hexane solvent in a rotary evaporator. With the solvent washing procedure, 100% efficiency was achieved.

**Figure 9:**
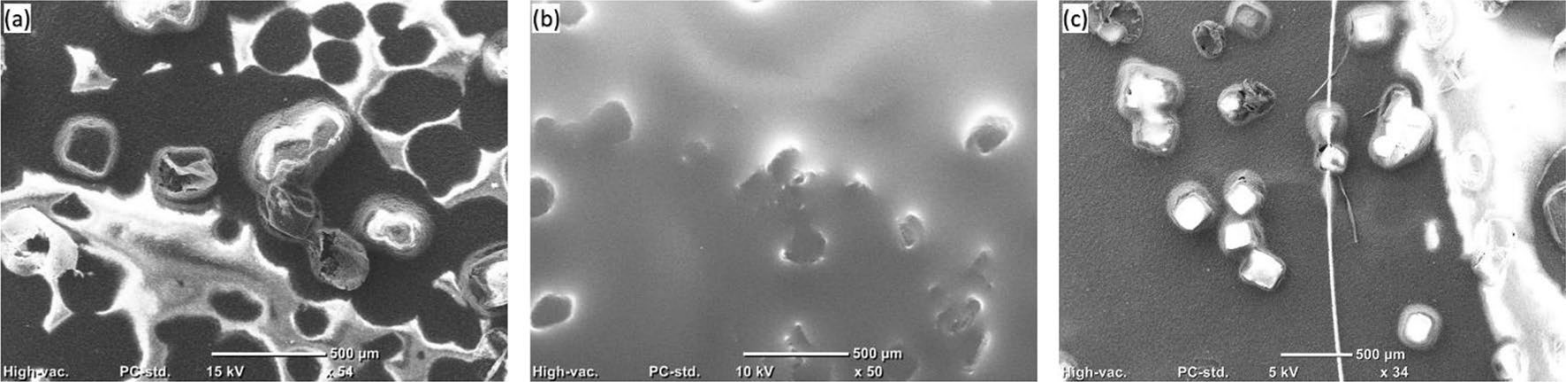
SEM images of oil sorbent (**a**) before oil sorption, (**b**) during oil sorption, (**c**) after oil sorption.

It was observed from the SEM investigations that the oil sorbent before and after oil sorption showed the retention of cavities without structural disintegration, see Fig. 9a. During the oil sorption, the sorbent absorbed and retained the oil on the surface and cavities get swollen showing the oil meniscus, see Fig. 9b. After 10 cycles, the oil was removed and washed with nonpolar solvent, the cavity structures were retained and the surface showed no damage, see Fig. 9c. And thus, these sorbents can be used for repeated times.

### Kinetics study

**Figure 10:**
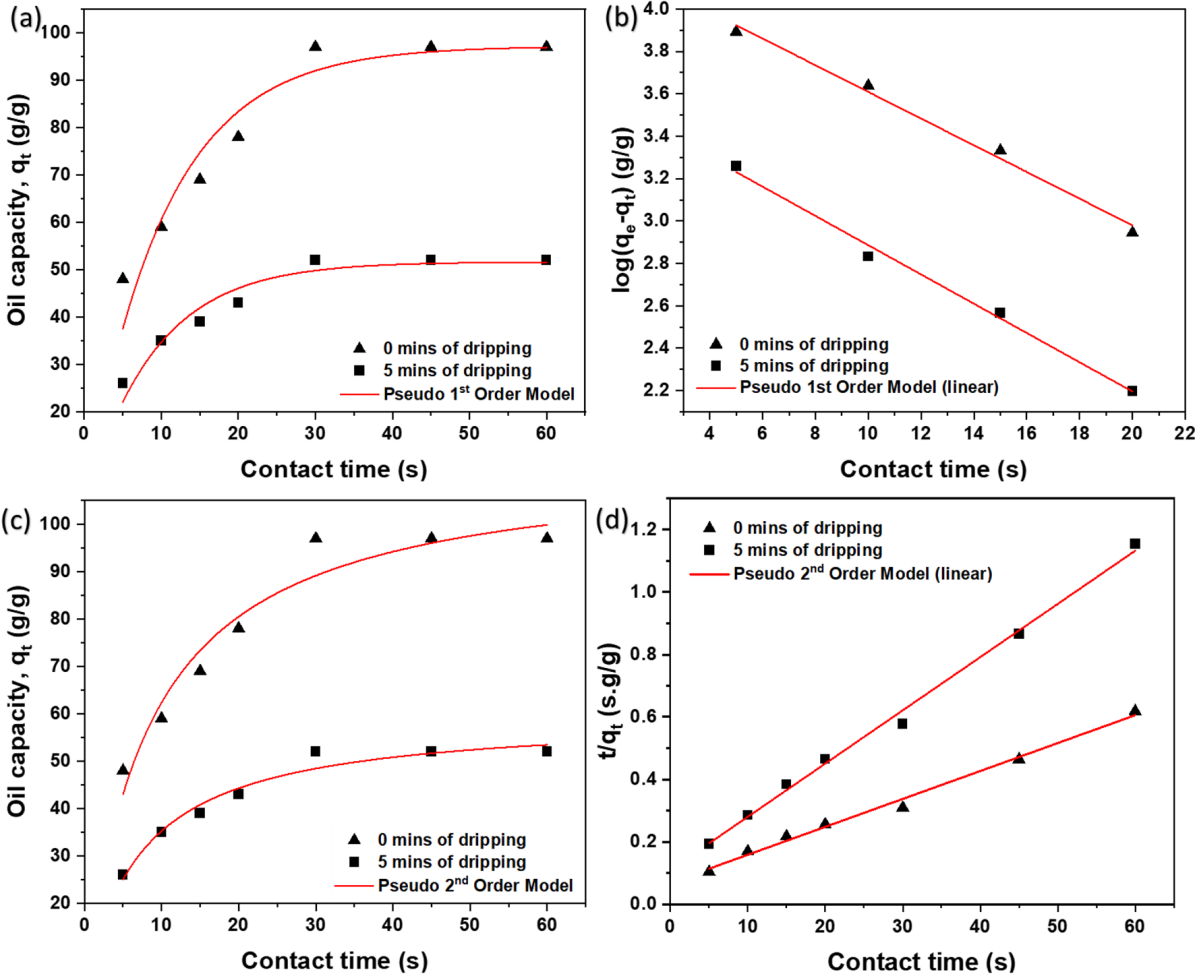
Modelling of sorption kinetics based on (**a**) non-linear form of Pseudo First-Order Model (**b**) linear form of Pseudo First-Order Model (**c**) non-linear form of Pseudo Second-Order Model and (**d**) linear form of Pseudo Second-Order Model.

In Fig. 10, both pseudo-first order (PFO) and pseudo-second order (PSO) models were used to model the uptake kinetics. The resulting plots were then used to determine the sorption constants, which are listed in Table 2. The sorption capacity (qe) is a parameter that affects the value of ‘k’.

**Table 2:** Fitted parameter values of kinetic models.

Kinetics model	0 min of dripping (immediate measurements)				5 min of dripping (measurements at equilibrium)			
	Measured q_e_ (g/g)	q_e_ (g/g)	Rate constant, k	R^2^	Measured q_e_ (g/g)	q_e_ (g/g)	Rate constant, k	R^2^
PFO (non-linear)	97	97.16	0.22485	0.91758	52	51.64	0.25657	0.93595
PFO (linear)	97	69.30	0.14498	0.99098	52	35.73	0.15895	0.99317
PSO (non-linear)	97	113.51	0.00107	0.94523	52	59.59	0.00243	0.96597
PSO (linear)	97	111.81	0.00116	0.99221	52	58.66	0.00264	0.99547

Sorption kinetics of dripping at retention value in comparison with measurements immediately done after sorption showed higher rate constant. The value of qe will also decrease over time as loosely connected oil drips off from the sorbent sponge until equilibrium is reached with no more dripping. From the Table 2 it can be observed that the (q_e_) which is the sorption capacity at final stage is predicted in good agreement with PFO. Moreover, Fig. 10d shows good agreement of PSO for both the stages of sorption. But comparatively, it can be seen from Fig. 10a,c that PSO is a good fit at initial stage of sorption whereas PFO is in good agreement at the final stage of sorption.

Moreover, the term ‘t_h_,’ or half time, is used to measure the sorption rate, representing the time needed to reach half of the saturated sorption capacity. A low t_h_ value indicates fast uptake. The best-fitted correlation, as shown in Fig. 10c, corresponds to PSO and gives a t_h_ value of 8.23 s and 6.9 s for 0 and 5 min of dripping, respectively.

## Conclusions

Our work involved designing a sorbent with trimodal structure using recycled PP. The structure was made up of micropores, macro-voids, and 3D swellable cavities. We conducted experiments to identify the optimal conditions for achieving high porosity for effective sorption with sufficient tensile strength. Based on our findings, we determined that the ideal conditions were 160 °C and 20 min of processing time. Under these conditions, we were able to create a 7 µm sorbent film with 34% porosity and a tensile strength of 5 MPa.The contact angles for toluene, engine oil, and sunflower oil were found to be  < 1°, 16.1°, and 26.5°, respectively, allowing faster penetration. The improved oil retention capacity was attributed to the cohesion and adhesion forces between oil molecules in the sorbent's structure and surface, as well as the adhesive forces between the sorbent and oil. The sorbent reached its saturation within 30 s of contact time with oil, exhibiting super-fast reaction kinetics and following a pseudo-second order kinetic model. After oil sorption, desorption, and resorption, the as-prepared sorbent maintained its structural integrity and could be recycled with 96.5% efficiency for hundreds of cycles. Furthermore, oil extraction from the film was easily achievable without requiring chemical processing.

## Data Availability

The datasets used and/or analysed during the current study available from the corresponding author on reasonable request.
